# Parathyroidectomy improves cardiovascular risk factors in normocalcemic and hypercalcemic primary hyperparathyroidism

**DOI:** 10.1186/s12872-019-1093-4

**Published:** 2019-05-08

**Authors:** Selvihan Beysel, Mustafa Caliskan, Muhammed Kizilgul, Mahmut Apaydin, Seyfullah Kan, Mustafa Ozbek, Erman Cakal

**Affiliations:** 1 Department of Endocrinology and Metabolism, Ankara Diskapi Yildirim Beyazit Teaching and Research Hospital, Ankara, Turkey; 2 Department of Medical Biology, Baskent University, Ankara, Turkey; 3 Department of Endocrinology and Metabolism, Afyonkarahisar Saglik Bilimleri University, Afyonkarahisar, Turkey

**Keywords:** Normocalcemic primary hyperparathyroidism, Hypercalcemic primary hyperparathyroidism, Cardiovascular risk

## Abstract

**Background:**

Parathyroidectomy has ameliorated cardiovascular risk factors in patients with hypercalcemic primary hyperparathyroidism (PHPT), but the effect of parathyroidectomy on normocalcemic PHPT is not exactly known. This case-controlled study aimed to investigate the effect of parathyroidectomy on cardiovascular risk factors in patients with normocalcemic and hypercalcemic PHPT.

**Methods:**

Subjects with normocalcemic PHPT (*n* = 35), age- and sex-matched hypercalcemic PHPT (*n* = 60) and age- and sex-matched control (*n* = 60) were included. Cardiometabolic disorders were investigated with traditional cardiometabolic risk factors and the Framingham cardiovascular risk score (CRS) before and 6 months after parathyroidectomy.

**Results:**

Diabetes, dyslipidemia, hypertension, obesity, insulin resistance, osteoporosis, having fractures were similarly increased in the hypercalcemic and normocalcemic PHPT groups (*p* > 0.05) compared with the controls (*p* < 0.05). Blood pressures, glucose metabolism (glucose, insulin, HOMA-IR) and lipid profiles were similarly increased in the PHPT groups (*p* > 0.05) compared with the controls (*p* < 0.05). After parathyroidectomy, blood pressures, serum total cholesterol, and HOMA-IR were decreased in both PHPT groups (*p* < 0.05). CRS was lower in the controls (5.74 ± 3.24, *p* < 0.05). After parathyroidectomy, CRS was decreased in the normocalcemic (11.98 ± 10.11 vs. 7.37 ± 4.48) and hypercalcemic (14.62 ± 11.06 vs. 8.05 ± 7.72) PHPT groups. Increased blood pressures were independent predictors of serum iPTH.

**Conclusion:**

The normocalcemic and hypercalcemic PHPT groups had similarly increased cardiovascular risk factors, even independently of serum calcium. Parathyroidectomy ameliorated the increased cardiovascular risk factors in both normocalcemic and hypercalcemic PHPT.

## Background

Primary hyperparathyroidism (PHPT) is characterized by hypercalcemia and elevated parathyroid hormone (PTH) concentrations. Normocalcemic PHPT represents a special form of PHPT, with normal total serum calcium and ionized calcium concentrations in the presence of elevated PTH concentrations [1, 2]. In such cases, the following secondary conditions that cause elevated PTH should be ruled out: renal disease, vitamin D deficiency, renal hypercalciuria, calcium malabsorption, and thiazide or lithium usage [3]. Typically, normocalcemic patients with PHPT present with symptomatic complications such as osteoporosis and nephrolithiasis. Non-traditional manifestations of normocalcemic PHPT, namely cardiovascular and metabolic disorders, are not known by physicians who recognize PHPT by its symptomatic complications. The asymptomatic form of normocalcemic PHPT has also been defined in population-based screening programs [4, 5]. Hypercalcemic PHPT is associated with increased hypertension, dyslipidemia, obesity, impaired glucose intolerance, and diabetes mellitus [6, 7], as well as cardiovascular morbidity and mortality[1, 8–11]. Parathyroidectomy ameliorates those cardiovascular risk factors in patients with hypercalcemic PHPT [7–11]. However, the effect of parathyroidectomy on cardiovascular risk factors in patients with normocalcemic PHPT is not exactly known. In population-based studies, elevated calcium and PTH concentrations have been linked to increased cardiovascular risk [12–15]. There is lack of data on the effect of parathyroidectomy on cardiovascular risk factors in patients with normocalcemic PHPT [16]. This study aimed to investigate the effect of parathyroidectomy on cardiometabolic disorders in patients with normocalcemic and hypercalcemic PHPT.

## Methods

### Patients

Patients with PHPT treated in the Department of Endocrinology and Metabolism, Ankara Diskapi Training and Research Hospital, from 2013 to 2017, were recruited to this case-control study. Normocalcemic patients with PHPT (*n* = 35) and age- and sex-matched patients with hypercalcemic PHPT (*n* = 60), and age- and sex-matched control subjects (*n* = 60) were included. The controls subjects were selected from population-based screening programs. Controls subjects without diabetes and hyperparathyroidism were included. Subjects using anti-lipidemic drugs and antihypertensive drugs were not included. Most patients with normocalcemia were referred from the osteoporosis outpatient clinic.

In our report, 54 patients had normocalcemia with elevated PTH concentrations, and 19 patients who were normocalcemic and treated with bisphosphonates were excluded form study. Forty-two patients who were hypercalcemic and treated with bisphosphonates were excluded. Patients with hypercalcemic (*n* = 60) and normocalcemic PHPT (*n* = 35) who were not using bisphosphonates were included. We evaluated symptoms related with hypercalcemia (repeated nephrolithiasis, gastritis, polyuria, muscle weakness, osteoporosis or psychiatric disorders) and bone fractures.

Hypercalcemic crisis was defined as serum calcium ≥14 mg/dL. Patients with hypercalcemic crisis were not included. The diagnosis of hypercalcemic PHPT was based on elevated serum albumin-corrected calcium (> 10.5 mg/dL) with high serum iPTH concentrations (> 65 pg/mL). Normocalcemic PHPT was diagnosed in the presence of elevated serum iPTH concentrations (> 65 pg/mL) with normal serum albumin-corrected calcium (≤10.4 mg/dL) on two separate occasions. All patients had 25-hydroxyvitamin D (25(OH)vitamin D) concentrations above 30 ng/mL.

Secondary causes of hyperparathyroidism were excluded, including renal insufficiency (creatinine clearance < 90 mL/min), liver disease, significant hypercalciuria (urinary calcium > 350 mg per 24 h), thiazide or lithium use and other metabolic bone diseases. All patients with PHPT underwent localization studies such as neck ultrasonography and Tc99-sestamibi and/or single-photon emission-computed tomography (SPECT-CT) scintigraphy. All patients with PHPT underwent four gland explorations and the incision size was the same in the two groups. All operations were performed by a single surgical group. All patients had the following characteristics: biochemical and clinical diagnosis of PHPT, parathyroidectomy, successful parathyroidectomy as confirmed by normal post-parathyroidectomy serum calcium and iPTH concentrations and pathologic confirmation of PHPT. Patients with incomplete clinical and biochemical evaluation were eliminated. Diabetes, dyslipidemia, hypertension, obesity, fractures, smoking and insulin resistance were recorded. Medications including anti-lipidemics, antihypertensives, and oral antidiabetics/insulin were recorded. Each subject gave written informed consent in accordance with the Declaration of Helsinki, and this study was approved by Diskapi Teaching and Training Hospital Ethic Committee.

### Measurements

Cardiometabolic disorders were investigated through traditional cardiometabolic risk factors and the Framingham 10-year general cardiovascular risk score before and after parathyroidectomy. Anthropometric measurements and biochemical analysis were performed in all patients before surgery and 6 months after surgery. The waist-hip ratio and body mass index (BMI) was calculated. Office blood pressure (BP) was measured in each subject in the sitting position after 5 min of rest, provided that the arm was supported at the level of the heart. A cuff covered about 80% of the circumference of the upper arm with the lower edge 2. 5-3 cm above the elbow. Hypertension was confirmed by repeated measurements of systolic blood pressure (BP) > 140 mmHg and diastolic BP > 90 mmHg on two separate occasions. Serum calcium, phosphorus, intact parathyroid hormone (iPTH), 25(OH) vitamin D, alkaline phosphatase (ALP), glycated hemoglobin (HbA1c), total cholesterol, high-density lipoprotein cholesterol (HDL-C), low-density lipoprotein cholesterol (LDL-C), triglycerides (TG), glucose, and insulin were measured after overnight fasting. Patients performed a 24-hour urine collection for measurements of urinary calcium excretion. iPTH was measured using a radioimmunoassay method (DiaSorin Inc., Stillwater, USA). Serum total calcium was corrected for albumin using the following formula:

$$ \mathrm{albumin}-\mathrm{corrected}\ \mathrm{calcium}=\left[0.8\ \mathrm{x}\ \left(4-\mathrm{serum}\ \mathrm{albumin}\right)\right]+\mathrm{serum}\ \mathrm{calcium} $$

Diabetes and prediabetes were diagnosed as follows: diabetes HbA1c 6.5% or higher; prediabetes HbA1c 5.7 to 6.4%; normal less than HbA1c 5.7%. Insulin resistance was calculated using the homeostasis model assessment-insulin resistance (HOMA-IR) formula [17]:

$$ \left[\mathrm{fasting}\ \mathrm{plasma}\ \mathrm{insulin}\ \left(\upmu \mathrm{IU}/\mathrm{ml}\right)\ \mathrm{X}\ \mathrm{fasting}\ \mathrm{serum}\ \mathrm{glucose}\ \left(\mathrm{mg}/\mathrm{dl}\right)\right]/405 $$

The Framingham General Cardiovascular Risk Score (10-year risk) (CRS) was computed through the online interactive risk score calculator available on the Framingham Heart Study website [18]. The 10-year general CV risk profile based on the Framingham study consisted of a total of 7 items such as sex, age, systolic BP, HDL-C, total cholesterol, and whether the individual had diabetes or smoked.

### Statistical analysis

Statistical analysis was performed using the SPSS 18.0 (SPSS, Inc.) statistical software. Variables are presented as mean ± standard deviation (SD) or median (with interquartile range), percentage (%), odds ratios (*OR*) and 95% confidence intervals (*CI*). Normality was tested using the Kolmogorov-Smirnov and Shapiro-Wilk *W* test. The Chi-square test or Fisher’s exact test, where appropriate, was used for categorical variables. Student’s t-test was used for the analysis of continuous variables between the two groups. The paired-sample t-test was used for the two groups before and after parathyroidectomy. Backward logistic regression was performed: serum iPTH was defined as dependent variable and calcium, LDL-C, HOMA-IR, creatinine clearance, and systolic and diastolic BP were independent variables. Statistical significance was defined as a *p* < 0.05.

## Results

Sex and mean age were similar between the groups (*p* > 0.05). Some of the controls were newly diagnosed as having dyslipidemia (16.7%) and hypertension (15.0%) in population-based screening programs during the study. The rate of prediabetes was 8.7% among the controls according to HbA1c levels. The prevalence of patients with diabetes, dyslipidemia, hypertension, obesity, insulin resistance, osteoporosis, having fractures, and medications usage were similar between hypercalcemic and normocalcemic PHPT groups (*p* > 0.05). Serum calcium and iPTH concentrations and urinary calcium excretion were higher in patients with hypercalcemic PHPT compared with normocalcemic PHPT (*p* < 0.05). Blood pressures and creatinine clearance were similar between the PHPT groups (*p* > 0.05). Glucose metabolism (glucose, insulin, HOMA-IR) and lipid profiles were similar between the PHPT groups (*p* > 0.05). Cardiovascular risk score was lower in controls, but similar between the PHPT groups (5.74 ± 3.24, 11.98 ± 10.11, and 14.62 ± 11.07, respectively). The baseline characteristics of the groups before parathyroidectomy are shown in Table 1.

**Table 1:** Baseline characteristics of subjects in controls, normocalcemic PHPT and hypercalcemic PHPT group

Variables	Controls (*n* = 60) n (%)	Normocalcemic PHPT (*n* = 35) n (%)	Hypercalcemic PHPT (*n* = 60) n (%)	p 1^a^	p 2^b^	p 3^c^
Female	48 (80.0%)	29 (82.9%)	47 (78.3%)	0.759	0.815	0.652
Diabetes	–	8 (22.8%)	14 (23.3%)	–	–	0.869
Dyslipidemia	10 (16.7%)	22 (62.9%)	36 (60.0%)	**< 0.001**	**< 0.001**	0.835
Hypertension	9 (15.0%)	15 (42.9%)	38 (63.3%)	**< 0.001**	**< 0.001**	0.094
Smoking	10 (16.7%)	5 (14.3%)	12 (20.0%)	0.743	0.759	0.526
Obesity	26 (43.3%)	16 (45.7%)	32 (53.3%)	0.810	0.339	0.473
İnsulin resistance	13 (21.7%)	21 (60.0%)	41 (68.3%)	**0.002**	**0.001**	0.454
Nephrolithiasis	3 (0.05%)	9 (25.7%)	29 (48.3%)	**0.001**	**< 0.001**	**0.048**
Osteoporosis	–	8 (22.9%)	23 (38.3%)	–	–	0.064
Fractures	–	2 (5.7%)	6 (10.0%)	–	–	0.624
Subjects using medications (%)	–			–	–	
Antilipidemic drugs		14 (40.0%)	27 (45.0%)			0.864
Antihypertensive drugs		13 (37.1%)	33 (55.0%)			0.125
Oral antidiabetic/insulin drugs		7 (20.0%)	13 (21.6%)			0.898
Age (year)	52.49 ± 8.17	53.06 ± 8.76	52.11 ± 10.04	0.204	0.320	0.998
BMI (kg/m^2^)	29.31 ± 3.59	29.97 ± 3.73	31.09 ± 5.17	0.629	**0.009**	**0.025**
Waist-hip-ratio	0.92 ± 0.09	0.90 ± 0.06	0.90 ± 0.06	0.293	0.375	0.852
Diastolic BP (mmHg)	77.5 ± 5.4	82.3 ± 5.8	83.2 ± 7.6	**0.001**	**< 0.001**	0.563
Systolic BP (mmHg)	121.6 ± 9.2	136.6 ± 16.5	138.9 ± 16.4	**< 0.001**	**< 0.001**	0.545
iPTH (pg/ml)	46.1 ± 10.3	145.8 ± 40.1	188.5 ± 93.3	**< 0.001**	**< 0.001**	**0.010**
Albumin-corrected calcium (mg/dl)	8.78 ± 0.29	9.85 ± 0.30	11.25 ± 0.69	**< 0.001**	**< 0.001**	**< 0.001**
Phosphorus (mg/dl)	3.22 ± 0.44	2.98 ± 0.48	2.57 ± 0.41	0.082	**< 0.001**	0.064
ALP (IU/L)	70.09 ± 14.85	103.51 ± 40.82	107.00 ± 44.27	**< 0.001**	**< 0.001**	0.735
25 (OH) vitamin D_3_ (nmol/l)	32.94 ± 4.89	33.43 ± 3.18	32.06 ± 4.65	0.845	0.692	0.601
Glucose (mg/dl)	86.69 ± 5.49	93.11 ± 12.19	95.00 ± 14.80	**0.007**	**0.003**	0.567
İnsulin (μIU/ml)	9.18 ± 3.96	13.26 ± 6.19	13.33 ± 5.24	**0.002**	**< 0.001**	0.953
HOMA-IR	1.98 ± 0.90	3.06 ± 1.47	3.13 ± 1.25	**< 0.001**	**< 0.001**	0.821
Total cholesterol (mg/dl)	185.60 ± 21.22	210.09 ± 39.46	204.80 ± 37.98	**0.002**	**0.01**	0.564
LDL-C (mg/dl)	107.80 ± 17.61	129.20 ± 33.11	123.54 ± 30.33	**0.001**	**0.01**	0.451
HDL-C (mg/dl)	52.80 ± 7.68	49.17 ± 10.56	49.34 ± 11.29	0.104	0.136	0.945
TG (mg/dl)	125.00 ± 51.20	158.60 ± 61.78	159.57 ± 67.34	**0.016**	**0.018**	0.951
Creatinine clerance (ml/min/1.73 m^2^)	113.92 ± 10.85	112.89 ± 27.26	107.15 ± 28.37	0.834	0.198	0.394
Urinary calcium (mmol/dl)	.	218.69 ± 162.74	438.49 ± 186.99	–	–	**0.010**
Cardiovascular risk score	5.74 ± 3.24	11.98 ± 10.11	14.62 ± 11.06	**0.001**	**< 0.001**	0.251

^a^controls vs normocalcemic

^b^controls vs hypercalcemic

^c^hypercalcemic vs normocalcemic

Data are presented as mean ± SD

*Abbreviations*: *BP* blood pressure, *iPTH* intact parathyroid hormone, *25(OH) vitamin D* 25-hydroxyvitamin D3, *HDL-C* high-density-lipoprotein cholesterol, *LDL-C* low-density-lipoprotein cholesterol, *TG* triglycerides, *HOMA-IR* homestasis model assessment-insulin resistance index, *ALP* alkaline phosphatase, *PHPT* primary hyperparathyroidism

Bold represents the significant *p*-values

After parathyroidectomy, serum calcium, iPTH, and ALP concentrations decreased, whereas serum phosphorous was increased in both PHPT groups (*p* < 0.05). Systolic and diastolic blood pressure was decreased in both PHPT groups (*p* < 0.05). Serum total cholesterol and HOMA-IR was decreased in both PHPT groups (*p* < 0.05). The change in metabolic parameters before and after parathyroidectomy is shown in Table 2. Cardiovascular risk score was decreased after parathyroidectomy in the normocalcemic (11.98 ± 10.11 vs. 7.37 ± 4.48) and hypercalcemic (14.62 ± 11.06 vs. 8.05 ± 7.72) PHPT groups. After parathyroidectomy, patients with fractures had more severe cardiovascular risk score compared with patients without fractures (6.72 ± 4.92 vs. 14.75 ± 9.47, *p* = 0.010) (Table 3). Hypercalcemic (*OR* = 4.39, 95% CI: [2.51–8.53]; *p* < 0.001) and normocalcemic (*OR* = 3.16, 95% CI: [2.7 8-6.92]; *p* = 0.002) PHPT was associated with increased in cardiovascular risk score. Systolic blood pressure (*ß* = 1.081, 95% CI: [1.03–1.13]; *p* = 0.001) and diastolic blood pressure (*ß* = 1.192, 95% CI: [1.04–1.32]; *p* = 0.05) were independent predictors of serum iPTH values, but not associated with calcium values (Cox & Snell R^2^ = 0.40).

**Table 2:** Change in metabolic parameters after parathyroidectomy

Variables	Normocalcemic PHPT (*n* = 30)			Hypercalcemic PHPT (*n* = 50)		
	Pre-PTX	Post-PTX	P	Pre-PTX	Post-PTX	P
BMI (kg/m^2^)	29.97 ± 3.73	29.98 ± 3.65	0.372	31.09 ± 5.17	29.47 ± 5.61	0.532
Waist-hip-ratio	0.89 ± 0.05	0.88 ± 0.05	0.177	0.89 ± 0.06	0.88 ± 0.68	0.363
Diastolic BP (mmHg)	82.3 ± 5.8	79.36 ± 7.74	**0.040**	83.2 ± 7.6	77.01 ± 7.38	**0.008**
Systolic BP (mmHg)	136.6 ± 16.5	126.57 ± 16.57	**0.003**	138.9 ± 16.4	119.73 ± 12.95	**0.001**
iPTH (pg/ml)	145.8 ± 40.1	71.52 ± 21.50	**< 0.001**	188.5 ± 93.3	48.60 ± 18.97	**0.001**
Albumin-corrected calcium (mg/dl)	9.85 ± 0.30	8.91 ± 0.38	**< 0.001**	11.25 ± 0.69	9.02 ± 0.32	**0.001**
Phosphorus (mg/dl)	2.98 ± 0.48	3.20 ± 0.41	**0.002**	2.57 ± 0.41	3.21 ± 0.47	**0.001**
ALP (IU/L)	103.51 ± 40.82	86.89 ± 25.10	**< 0.001**	107.00 ± 44.27	84.33 ± 2.54	**0.001**
Glucose (mg/dl)	93.11 ± 12.19	86.36 ± 5.97	0.120	95.00 ± 14.80	82.26 ± 6.70	**0.009**
İnsulin (μIU/ml)	13.26 ± 6.19	11.47 ± 3.08	**0.006**	13.33 ± 5.24	10.83 ± 4.62	0.125
HOMA-IR	3.06 ± 1.47	2.57 ± 0.81	**0.024**	3.13 ± 1.25	2.25 ± 0.92	**0.031**
Total cholesterol (mg/dl)	210.09 ± 39.46	204.06 ± 41.92	**0.002**	204.80 ± 37.98	185.97 ± 28.62	**0.017**
LDL-C (mg/dl)	129.20 ± 33.11	127.73 ± 36.71	**0.004**	123.54 ± 30.33	109.41 ± 16.65	0.094
HDL-C (mg/dl)	49.17 ± 10.56	49.94 ± 7.96	**0.046**	49.34 ± 11.29	49.01 ± 11.06	0.138
TG (mg/dl)	158.60 ± 61.78	137.84 ± 61.55	**0.019**	159.57 ± 67.34	132.93 ± 53.95	0.172
Creatinine clerance (ml/min/1.73 m^2^)	112.89 ± 27.26	123.36 ± 31.44	0.777	107.15 ± 28.37	121.75 ± 39.10	0.198
Cardiovascular risk score	11.98 ± 10.11	7.37 ± 4.48	**0.001**	14.62 ± 11.06	8.05 ± 7.72	**0.005**

Data are shown as mean ± standard deviation (means±SD)

*Abbreviations*: *BP* blood pressure, *iPTH* intact parathyroid hormone, *25(OH) vitamin D* 25-hydroxyvitamin D3, *HDL-C* high-density-lipoprotein cholesterol, *LDL-C* low-density-lipoprotein cholesterol, *TG* triglycerides, *HOMA-IR* homestasis model assessment-insulin resistance index, *ALP* alkaline phosphatase, *PHPT* primary hyperparathyroidism

Bold represents the significant *p*-values

**Table 3:** Metabolic parameters of subjects with and without fractures before and after parathyroidectomy

	Subjects without fractures	Subjects with fractures	p
Pre-parathyroidectomy			
Cardiovascular risk score	12.71 ± 10.88	17.85 ± 7.04	0.199
Diastolic BP (mmHg)	81.93 ± 6.29	89.50 ± 3.50	**0.001**
Systolic BP (mmHg)	137.37 ± 16.61	141.010 ± 11.23	0.551
iPTH (pg/ml)	163.71 ± 71.17	194.01 ± 98.26	0.282
Calcium (mg/dl)	10.62 ± 0.77	11.06 ± 0.71	0.136
Phosphorus (mg/dl)	2.69 ± 0.44	2.42 ± 0.49	0.119
ALP (IU/L)	98.76 ± 27.35	155.63 ± 88.58	**< 0.001**
Glucose (mg/dl)	93.92 ± 12.57	95.13 ± 20.42	0.814
İnsulin (μIU/ml)	13.90 ± 5.65	8.57 ± 3.49	**0.012**
HOMA-IR	3.22 ± 1.33	2.09 ± 1.16	**0.026**
Total cholesterol (mg/dl)	206.67 ± 39.21	213.45 ± 34.48	0.643
LDL-C (mg/dl)	126.19 ± 32.69	127.75 ± 23.59	0.897
HDL-C (mg/dl)	49.63 ± 11.04	49.38 ± 9.39	0.429
TG (mg/dl)	155.95 ± 57.35	196.63 ± 101.29	0.092
Post-parathyroidectomy			
Cardiovascular risk score	6.72 ± 4.92	14.75 ± 9.47	**0.010**
Diastolic BP (mmHg)	77.43 ± 7.33	85.00 ± 6.48	0.059
Systolic BP (mmHg)	123.83 ± 14.04	121.51 ± 25.43	0.779
iPTH (pg/ml)	61.46 ± 23.57	61.04 ± 23.59	0.971
Calcium (mg/dl)	8.94 ± 0.35	9.06 ± 0.44	0.552
Phosphorus (mg/dl)	3.26 ± 0.36	2.77 ± 0.69	**0.034**
ALP (IU/L)	81.04 ± 20.18	121.56 ± 27.24	**0.001**
Glucose (mg/dl)	85.40 ± 6.01	78.25 ± 7.88	**0.038**
İnsulin (μIU/ml)	11.81 ± 3.44	6.53 ± 3.26	**0.007**
HOMA-IR	2.55 ± 0.80	1.47 ± 0.70	**0.015**
Total cholesterol (mg/dl)	194.49 ± 37.62	207.00 ± 37.97	0.537
LDL-C (mg/dl)	119.70 ± 32.10	119.25 ± 19.01	0.978
HDL-C (mg/dl)	49.30 ± 9.23	51.25 ± 11.17	0.757
TG (mg/dl)	133.36 ± 54.56	153.03 ± 84.59	0.530

Data are shown as mean ± standard deviation (means±SD)

*Abbreviations*: *BP* blood pressure, *iPTH* intact parathyroid hormone, *25(OH) vitamin D* 25-hydroxyvitamin D3, *HDL-C* high-density-lipoprotein cholesterol, *LDL-C* low-density-lipoprotein cholesterol, *TG* triglycerides, *HOMA-IR* homestasis model assessment-insulin resistance index, *ALP* alkaline phosphatase, *PHPT* primary hyperparathyroidism

Bold represents the significant *p*-values

## Discussion

The normocalcemic and hypercalcemic PHPT groups had similarly increased cardiovascular risk factors including blood pressures, glucose metabolism, and lipid profiles as well as cardiovascular risk scores compared with the controls. Parathyroidectomy improved the increased cardiovascular risk factors including blood pressures, total cholesterol, and HOMA-IR as well as cardiovascular risk scores in patients with normocalcemic and hypercalcemic PHPT. An increased serum PTH concentration was found as a positive predictor of increased blood pressures, even independent of serum calcium.

Normocalcemic PHPT is typically recognized through symptoms such as osteoporosis and nephrolithiasis, and the potential cardiometabolic effects of this disease are not known. Hypercalcemic PHPT was reported to indicate an increased risk for cardiovascular diseases [19, 20]. Increased cardiovascular morbidity and mortality was reported in hypercalcemic PHPT [8–11]. Obesity [19], hypertension [8], hyperlipidemia [8], and diabetes [21] is higher in PHPT compared with the normal population, a potential cause for cardiovascular diseases [6, 22, 23]. Successful surgery for hyperparathyroidism is followed by improvements in hypertension, impaired glucose metabolism, dyslipidemia, and even in cardiovascular dysfunction [24, 25]. It is believed that elevated serum calcium and PTH concentrations might be directly or indirectly associated with increased cardiovascular risk [12–14]. However, there is still a lack of data on the potential cardiometabolic effects of normocalcemic PHPT after parathyroidectomy.

In present study, the prevalence of disease such as hypertension, diabetes, dyslipidemia, obesity, and insulin resistance were similarly increased in both patients with normocalcemic and hypercalcemic PHPT. Cardiometabolic risk factors including blood pressures, glucose metabolism and lipid profiles as well as cardiovascular risk score increased similarly in both patients with normocalcemic and hypercalcemic PHPT. Parathyroidectomy improved increased cardiovascular risk factors including systolic and diastolic BP, serum total cholesterol, and HOMA-IR, as well as cardiovascular risk scores in both patients with hypercalcemic and normocalcemic PHPT. Ozturk et al. found similar metabolic disorders including hypertension and glucose intolerance in patients with normocalcemic and hypercalcemic PHPT [26]. Traditional cardiovascular risk factors including hypertension, hyperlipidemia, and impaired glucose tolerance were found to be similar in normocalcemic and hypercalcemic PHPT, whereas cardiovascular and cerebrovascular diseases were found higher in patients with hypercalcemic PHPT [16]. Although fasting blood glucose was found to be elevated in patients with normocalcemic PHPT, insulin resistance, lipid profiles, and blood pressures in those patients was similar to the controls [27, 28]. Chen et al. observed that normocalcemic PHPT exhibited increased systolic and diastolic BP compared with the controls [29]. Increased PTH concentration was associated with increased fatal and non-fatal cardiovascular events [30]. Increased PTH was linked to increased cardiovascular morbidity and mortality, even in the absence of PHPT disease [20, 30, 31]. Increased PTH concentration was considered to be a potential marker of increased cardiovascular risk, even in the presence of normal calcium values [20, 31].

In the present study, increased serum PTH concentration acted as a positive predictor of increased systolic and diastolic BP. Hagström et al. reported improvements in dyslipidemia following parathyroidectomy in asymptomatic mild PHPT [9]. A positive correlation between increased PTH level and hypertension was observed [32–35]. Increased serum PTH concentrations were reported as a predictor of increased systolic BP [32]. Parathyroidectomy improved increased systolic BP in mild PHPT [8]. Several mechanisms may explain the association between serum PTH and blood pressure. PTH might directly or indirectly cause increased aldosterone secretion by activating the renin-angiotensin system or it might cause increased vessel wall thickness by triggering the proliferation of vascular smooth muscle cells. The bidirectional interplay between aldosterone and PTH leads to an increased risk of developing metabolic and cardiovascular diseases [36]. Hyperparathyroidism is involved in increased cardiovascular risk; however, other factors including PTH receptors, cardiomyocytes [37], endothelial cells, and vascular smooth muscles are possibly involved in the increased cardiovascular risk.

Elevated PTH concentrations should also be considered beyond calcium and phosphate dysregulation when explaining cardiovascular diseases [30, 36]. Chronic PTH elevation was correlated with coronary microvascular dysfunction, cardiac hypertrophy, impaired glucose and lipid mechanisms, arterial hypertension, endothelial dysfunction, and subclinical aortic valve calcification [1, 20, 38–40]. Such elevation might also be associated with pro-inflammatory protease and cytokine upregulation [30]. Elevated PTH concentrations were associated with adiposity and increased inflammatory cytokines in circulation (such as monocyte chemoattractant protein-1 and leptin) [40–42].

This case-control study and sample size was relatively small. These are limitations of this study.

## Conclusion

The present study observed that normocalcemic PHPT and hypercalcemic PHPT were had similarly increased cardiovascular risk factors, even independent of serum calcium. The normocalcemic and hypercalcemic PHPT groups had similarly increased cardiovascular risk factors including blood pressures, glucose metabolism, and lipid profiles, as well as cardiovascular risk scores compared with controls. Parathyroidectomy ameliorated increased cardiovascular risk factors including blood pressures, total cholesterol, and HOMA-IR, as well as cardiovascular risk scores in patients with normocalcemic and hypercalcemic PHPT. Serum PTH acted as a positive predictor of increased blood pressures. Further studies are needed to examine the effect of parathyroidectomy on non-traditional manifestations of normocalcemic PHPT. Such studies will explain the potential mechanism between serum PTH and cardiometabolic disorders.

## Abbreviations

25(OH) vitamin D_3_: 25-hydroxyvitamin D_3_
ALP: Alkaline phosphatase
BP: Blood pressure
CRS: Cardiovascular risk score
HDL-C: High-density-lipoprotein cholesterol
HOMA-IR: Homeostasis model assessment-insulin resistance index
iPTH: Intact parathyroid hormone
LDL-C: Low-density-lipoprotein cholesterol
PHPT: Primary hyperparathyroidism
TG: Triglycerides
